# Acute effects of electronic cigarette smoking on ventricular repolarization in adults

**DOI:** 10.4314/ahs.v20i4.33

**Published:** 2020-12

**Authors:** Vahit Demir, Siho Hidayet, Yaşar Turan, Hüseyin Ede

**Affiliations:** 1 Department of Cardiology, Medical Faculty of Bozok University, Yozgat, Turkey; 2 Department of Cardiology, Medical Faculty of Inonu University, Malatya, Turkey

**Keywords:** Electronic cigarette, ventricular repolarization, Tp-e interval, Tp-e/QT ratio

## Abstract

**Background:**

Electronic cigarette (e-cigarette) use is constantly increasing. However, the association between e-cigarette use and ventricular arrhythmia is unknown. Thus, in this study, we aimed to evaluate the markers of ventricular repolarization such as QT interval, corrected QT (QTc), QT dispersion (QTd), peak-to-end interval of the T wave (Tp-e), corrected Tp-e and Tp-e/QT ratios in e-cigarette users.

**Methods:**

The study population consisted 36 e-cigarette users and 40 healthy subjects. Ventricular repolarization parameters were obtained from 12-lead resting electrocardiogram. Ventricular repolarization parameters of the groups were compared.

**Results:**

Basal demographic and laboratory data were similar in both groups. According to the electrocardiographic parameters, the Tp-e interval, corrected Tp-e, and Tp-e/QT ratio were significantly higher in individuals using e-cigarettes than in control subjects [74.9±6.4 milliseconds (ms) vs. 80.1±4.1ms, <0.001; 82.9±7.5 ms vs. 87.8±6.3 ms, p=0.003; 0.20±0.01 vs. 0.21±0.01, p=0.002; respectively].

**Conclusion:**

This is the first study to show the disruption of ventricular repolarization properties in e-cigarette users. E-cigarette use in terms of public health leads to augmentation of transmural dispersion of repolarization, which may be potential indicator of ventricular arrhythmogenesis.

## Introduction

The effects of conventional cigarette on cardiovascular system have been extensively studied with numerous studies in adults. It is known that smoking is one of the main causes of cardiovascular events and causes ventricular arrhythmias and sudden cardiac deaths^1^. In recent years, electronic cigarettes (e-cigarette) have been launched as an auxiliary method for cessation of smoking by being claimed to be less harmful. Therefore, the frequency of e-cigarette use demonstrated a serious increase every year. E-cigarette is a source of nicotine designed as a pen or an accessory that gives the sense of smoking with an appearance similar to conventional cigarette^2^. Although e-cigarette is thought to be less harmful than normal cigarette, such thought does not implicate the safety of e-cigarette use^3^, ^4^.

Electrocardiography (ECG) parameters have been recommended to be used in cardiovascular risk screening independently from conventional cardiovascular risk factors in middle age group^5^. Various methods such as ventricular repolarization, QT interval, QT dispersion (QTd) and transmural dispersion of repolarization have been used for this purpose. Regional QT interval changes measured on ECG reflect the altered regional ventricular repolarization. Although the difference of maximum and minimum QT interval between derivations has been defined as QT dispersion (QTd), this may not directly reflect the dispersion of repolarization. Because QTd can be affected by the changes in T-wave shape and errors in QT interval measurements^6^. Abnormalities in ventricular repolarization increases sensitivity to ventricular arrhythmias. Recent studies stated that the distance (Tp-e) between the part where T-waves peak (p) and ends (e), as measured in surface ECG, may better reflect the global dispersion of repolarization^7^. Increased Tp-e interval may be a useful marker to demonstrate ventricular arrhythmia and cardiovascular mortality. Tp-e interval is affected by the changes in the heart rate. Therefore, Tpe/QT ratio is a better indicator of ventricular repolarization^7^. QT time, Tp-e interval, Tp-e/QT ratio are ECG variables with demonstrated importance in the estimation of arrhythmia and mortality development in various heart diseases^8^–^11^.

There is a lack of data on the long-term effects of e-cigarettes on the cardiovascular system. However, in studies examining the acute effects of e-cigarettes, it has been shown that e-cigarette causes potential cardiovascular toxicity in relation to increased heart rate, blood pressure and systemic oxidative stress^4^. This has led to the necessity for investigating the adverse potential effects of e-cigarette use on cardiovascular health outcomes. There is no study in the literature that examines the effects of e-cigarette on the parameters of ventricular repolarization.

The purpose of this study was to evaluate the acute effects of e-cigarette use on ventricular repolarization parameters [QT interval, corrected QT (QTc), QT dispersion (QTd), peak-to-end interval of the T wave (Tp-e), corrected Tp-e and ve Tp-e/QT ratios]

## Material and Method

This cross-sectional, prospective study was carried out at the Department of Cardiology, Yozgat Bozok University Faculty of Medicine, Turkey. Between June 2017 and June 2019, 36 e-cigarette users who were followed up at the cardiology outpatient clinic and 40 healthy subjects with similar age and sex and did not use any tobacco and nicotine-containing products were included in the study (76 individuals in total). Subjects aged between 18 and 55 years old who did not have any accompanying diseases, did not smoke conventional cigarettes and had been using e-cigarette for at least 6 months formed the e-cigarette group.

Individuals who consumed caffeine-containing beverages or stimulating substances within 12 hours prior to ECG, alcohol users (>112 gram/week ethyl alcohol intake in men, >72 gram/week in women), patients with acute infections, chronic inflammatory disease, diabetes mellitus or hypertension, patients with diagnosed coronary arteri disease (those with ischemia proof in stress test or >50% stenosis in any main vessel during coronary angiography or those with history of coronary revascularization), patients with dysrhythmia (atrial fibrillation, sustained atrial arrhythmia, those with >500 premature ventricular complex/24 hour in Holter or presence of a PVC on index resting ECG), electrolyte imbalance (serum potassium <4 mEg/L or serum magnesium <0.8 mmol/L) and patients whose ventricular repolarization parameters couldn't be calculated due to technical reasons were excluded from the study. Patients with a history of smoking in the last six months were excluded from the study.

Variable mode third generation e-cigarette users were assigned to the e-cigarette group. In the e-cigarette group, e-liquid kits containing medium to high density nicotine (16–21 mg/ml) were used for the device. Separate settings were used for e-cigarette users. Subjects were recommended to use the e-cigarette for a total of 25 puffs and 5 minutes in accordance with the manufacturer's instructions for use. Again in accordance with the manufacturer's recommendations, each breath was recommended to last for approximately 2–3 seconds. In accordance with the manufacturer's instructions, it was recommended that the battery be fully charged before use.

As the purpose of the study was to examine the acute effects of e-cigarette exposure on ventricular repolarization, standard 12-lead surface electrocardiography of the participants were obtained by using Nihon Kohden, Tokyo, Japan ECG device within 10 minutes following the use of nicotine-containing e-cigarette. Body mass index (BMI) was calculated by dividing body weight by the square of the neck (kg/m^2^). Transthoracic echocardiographic examination was performed with Philips Affinity 50 echocardiography device (Philips Healthcare, the Netherlands) according to the recommendations of American Society of Echocardiography. Left ventricular ejection fraction (LVEF) was calculated using the modified Simpson method. Blood samples for fasting blood glucose, whole blood count, fasting lipidrofile, serum electrolytes (potassium, magnesium, calcium) and serum creatinine levels were obtained between 8:00–10:00 in the morning after at least eight hours of fasting.

### Electrocardiographic Evaluation

**Figure 1:**
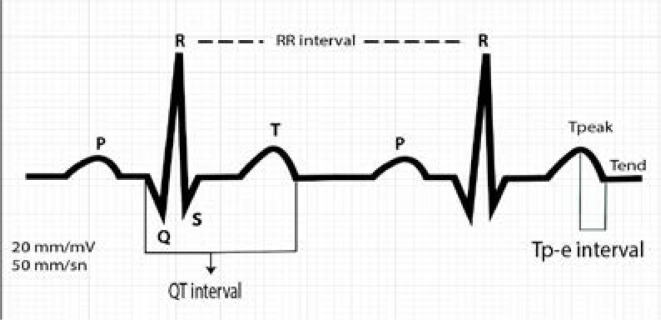
Demonstration of RR, QT and Tp-e intervals in electrocardiogram

Twelve-lead ECGs were obtained, at 20 mm/mV amplitude and 50 mm/s rate with standard lead positions in a supine position. Tp-e intervals were measured manually with calipers and magnifying glass to reduce the error rate. The QT interval was defined as the distance between the start of QRS and the last point at which the T wave intersects the isoelectric line. The TP-e interval was defined as the distance between the peak and the end of the T wave (Figure 1). TP-e interval measurement was performed using precordial surface ECG device in supine position. After normal sinus rhythm was observed by two independent cardiologists who were blinded to clinical details, the mean values of the measurements were recorded. Heart rate, RR interval and QT was evalauted by using leads and the mean results were calculated by using three consecutive cycles^10^. If T-wave amplitude is <1.5 and there is U-wave, then that lead was excluded from the analysis. QT maximum (QTmax) and QT minimum (QTmin) was calculated in all leads of 12-lead ECG. QT dispersion (QTd) was defined as the interval of QTmax-QTmin. Corrected QT (QTc) was calculated by using Bazzett formula 12. Tp-e/QT and Tp-e/QTc ratios were calculated based on the obtained values. The Tp-Te/QT ratio was defined as Tp-Te in lead V5 divided by QT interval in the same lead. The intra- and inter-observer variability was 4.3% and 4.9%, respectively.

The study protocol was approved by the local Ethics Committee (Approval no:2017-KAEK-189-2017.08.24-12) and the study protocol was explained to each patient and the patients who gave the written consent were included in the study.

## Statistical Analysis

Shapiro Wilk test was used to evaluate the distribution of normality of continuous variables. Statistical analysis of clinical data between two groups was analyzed by using Student t Test for normally distributed parameters and Mann Whitney U test for parameters without normal distribution. For correlations, Pearson or Spearman correlation coefficient was used. Chi-square test was used for categorical variables. Statistical analyses were performed on SPSS 20 (SPSS/IBM, Chicago, IL, USA) software, and p<0.05 was considered as the statistical significance level.

## Results

The mean age of e-cigarette users was 39.1±11.4 years and mean age of control subjects was 41.7±10.1 years. Clinical characteristics, laboratory parameters and left ventricular ejection fraction between the two groups are summarized in Table 1. There was no statistically significant difference between e-cigarette users and control subjects in term of sex, age, blood pressure, BMI and LVEF. The ECG characteristics of the groups, including heart rate, RR interval, Tp-e interval, corrected Tp-e interval, QTmin, QTmax, Corrected QTmin, Corrected QTmax, mean QTc, Tp-e/QT ratio, Tp-e/mean QTc ratio and QTd, are presented in Table 2.

**Table 1:** Baseline characteristics and echocardiographic and laboratory parameters

	Control Subjects (n=40)	E-Cigarette User (n=36)	P-value
Age, (Years)	39.1±11.4	41.7±10.1	0.289
Gender, (Female/Male)	10/30	8/28	0.695
BMI, (kg/m^2^)	26.0±3.4	27.3±5.8	0.344
SBP, (mmHg)	114.9±9.2	116.2±12.2	0.534
DBP, (mmHg)	71.9±7.7	72.4±9.2	0.804
FBG, (mg/dL)	96±12.1	96.9±12.3	0.447
Creatinine, (mg/dL)	0.84±0.13	0.84±0.14	0.996
Total Cholesterol, (mg/dL)	191.6±32.7	191.1±32	0.948
Triglyceride, (mg/dL)	146.5±55.4	164.4±86.7	0.655
HDL-cholesterol, (mg/dL)	48.3±9.7	45.3±10.9	0.216
LDL-cholesterol, (mg/dL)	112.6±29.6	110±25.9	0.775
Hemoglobin, (g/dL)	14.6±1.7	14.4±1.3	0.385
Leucocytes (10^3^/mm^3^)	7.1±1.5	6.5±1.7	0.099
Platelets (10^3^/mm^3^)	263.7±38.3	261.3±57.7	0.344
LVEF (%)	61.8±2.7	61.1±3	0.579

Expressed as Mean ± Standard Deviation, p values were compared by Student' T-test or chi-square test as appropriate, p<0.05 significant.

BMI: body mass index, SBP: Systolic blood pressure, DBP: Diastolic blood pressure, FBG: Fasting blood glucose, HDL: High-density lipoprotein; LDL: Low-density lipoprotein, LVEF: Left ventricular ejection fraction

**Table 2:** Electrocardiographic parameters of the study population

	Control subjects (n=40)	E-Cigarette User (n=36)	P-value
Heart rate (Bpm)	72.5±9.8	76±9.1	<0.001
RR interval (ms)	0.842±0.1	0.806±0.2	0.004
Tp-e (ms)	74.9±6.4	80.1±4.1	<0.001
Corrected Tp-e (ms)	82.9±7.5	87.8±6.3	0.003
QT minimum (ms)	352.8±20.6	360.8±17.3	0.060
QT maximum (ms)	378.8±24.6	388.6±16.8	0.040
Corrected QT minimum (ms)	389.9±20.9	394.9±19.7	0.280
Corrected QT maximum (ms)	418.6±25.3	425.1±20.4	0.227
Mean Corrected QT (ms)	404.3±22	409.4±19.7	0.291
QT dispersion (ms)	25.9±13.6	27.9±10.9	0.183
Corrected QT dispersion (ms)	28.7±14.9	30.6±12.4	0.268
Tp-e/QT (ms)	0.20±0.013	0.21±0.011	0.002
Tpe/meanQTc (ms)	0.18±0.016	0.20±0.014	0.003

Expressed as Mean ± Standard Deviation. Bpm: Beats per minute, Tp-e: Peak-to-end interval of the T wave

Baseline heart rate, RR interval was statistically significantly higher in the e-cigarette group. In the e-cigarette user group, Tp-e and Corrected Tp-e interval were significantly prolonged compared to the control subjects (80.1±4.1 milliseconds (ms) vs. 74.9±6.4 ms, p<0.001; 87.8±6.3 ms vs. 82.9±7.5 ms, p = 0.003, respectively). Both Tp-e/QT ratio and Tp-e/QTc ratio were found to be statistically higher in the e-cigarette users group than the healthy control group. (0.21±0.011 ms vs. 20±0.013 ms, p = 0.002; 0.20±0.014 ms vs. 0.18±0.016 ms, p = 0.003, respectively), (Table 2). There was no difference in terms of the other ECG parameters between the groups. E-cigarette users had been smoking for 58.2±8.4 months (range 6 to 94 months). There was no correlation between Tp-e interval, or Tpe/QT and Tpe/QTc ratio and the duration of e-cigarette use. Furthermore, there was no significant correlation between age, BMI, blood pressure with Tp-e interval, Tp-e/QT ratio, and Tp-e/QTc ratio.

## Discussion

The major new finding of this study was the determination of statistically significantly increased Tp-e interval, corrected Tp-e and Tp-e/QT ratio in e-cigarette users compared to healthy control subjects. These results provide important evidence on adverse effects of e-cigarette use on ventricular repolarization parameters in the acute stage. This is also the first study to report an increase in the Tp-e interval, Tp-e/QT ratio and Tp-e/QTc ratio among e-cigarette smokers.

E-cigarettes are battery-powered devices for the dissolution of nicotine and other aromas (coffee, mint or fruity tobacco, etc.) in the liquid consisting of propylene glycol and/or vegetable glycerol and transmission of nicotine in the vapor of this liquid mixture. The most commonly used solvents for nicotine are vegetable glycerol and propylene glycol. E-liquid solution heated by the atomized in the e-cigarette cartridge creates an aerosol mimicking the cigarette smoke^13^, ^14^. Other components of the solution include water, ethanol and various additives. The amounts of these substances in the cartridge vary among e-cigarette brands. Moreover, very high, high, medium, low and zero (nicotine-free) forms are available based on their nicotine content 4. The nicotine content in e-cigarettes is determined by manufacturers and varies between brands and even different models of the same brand. Nicotine content in an e-cigarette liquid ranges between 0–36.6 mg 3. E-cigarettes have the potential to contain nicotine equal or more than conventional tobacco cigarettes^14^.

Ilgenli et al. included 24 long-term smokers (mean age: 40 ± 5 years) and 23 non-smokers (mean age: 42 ± 10 years) in their study where the effects of conventional smoking on ventricular repolarization were examined. Basic clinical and echocardiographic data showed no significant difference between smokers and nonsmokers. QT interval and QTc interval was similar in both groups, however, Tp-e interval (78.9 ± 7.3 vs 85.3 ± 10.7, p = 0.02), Tpe-/QT ratio (0.21 ± 0.02 vs 0.25 ± 0.03, p = 0.001), Tpe/QTc ratio (0.20 ± 0.02 vs 0.23 ± 0.03, p = 0.001) parameters were found to be higher in smokers in comparison to non-smokers^15^. In addition to these parameters (Tp-e interval and Tp-e/QT, Tp-e/QTc ratios), Tasoler et al. demonstrated increased QT and QTd values in their study 16. And Kayali et al. showed that smoking had adverse effects on Tp-e interval and Tp-e/QT, Tp-e/QTc ratios in adolescents aged 16–19 years. Similar to conventional cigarette, Tp-e interval and Tp-e/QT, Tp-e/QTc ratios were statistically significantly higher among the e-cigarette users in our study. However, no statistically significant difference was found in terms of QTc and QTd. Based on our current knowledge, this is the first study to determine Tp-e interval and Tp-e / QT, Tp-e / QTc changes in adult e-cigarette users.

It is clearly known that nicotine released to the blood circulation during smoking increases plasma catecholamines, arterial blood pressure, and heart rate. All these changes can have an arrhythmogenic impact on heart by increasing the workload and oxygen need of myocardium^17^. Moreover, e-cigarette use increases heart rate and blood pressure faster than conventional cigarettes In twenty healthy non-smoking volunteers, 10 minutes of e-cigarette containing nicotine (18 mg) use significantly increased arterial pressure and heart rate^18^. In another study with a double blind design and 17 healthy subjects, the acute effects of inhaling the aerosol of e-cigarette (with and without nicotine) on vascular and pulmonary functions, nicotine e-cigarette aerosol caused a significant increase in blood pressure, heart rate and arterial stiffness^19^. This study demonstrates that inhaled e-cigarette aerosol with nicotine has a significant effect on vascular functions. In our findings, the heart rate was increased in the e-cigarette use group in accordance with the literature. However, arterial blood pressure increase was not consistent with the literature. This may be due to short-term resting of patients after acute e-cigarette use, and that their ECG measurements were taken first.

The duration of action potential is longer than other myocytes in medium myocardial M cells. Earliest repolarization occurs in epicardial cells. Epicardial action potential is represented with the peak of T-wave in ECG and the end of myocardial action potential is represented with the end of the T wave. Therefore, Tp-e interval is an indicator of transmural depolarization^20^, ^21^. The prolongation of TP-e interval has been previously demonstrated in Brugada syndrome, long QT syndrome, hypertrophic cardiomyopathy, in conventional smokers, patients with obstructive sleep apnea and Behcet's disease^11^, ^15^, ^21^–^23^. Prolongation of Tp-e interval while QTc is normal has been found to be associated with sudden cardiac death 24, 25. In recent years, Tp-e interval and Tp-e/QT ratio have become new electrocardiographic indices of abnormal dispersion of ventricular repolarization^26^. Moreover, it has been claimed that these parameters can be electrocardiographic markers of ventricular arrhythmogenesis and sudden death^27^, ^28^. Zumhagen et al.^29^ reported that Tp-Te/QT ratio evaluation in patients with Brugada syndrome could potentially be useful for the identification of life-threatening arrhythmias.

In most countries, the sale of nicotine-containing e-cigarettes is not permitted, but the sale of nicotine-free e-cigarettes is common and nicotine can be added to these later. When e-cigarette smoking characteristics are examined in terms of age groups, it is reported that it is preferred among young population.^13^, ^30^ Our study population consisted of young adults. The fact that e-cigarette is associated with the electrocardiographic markers of ventricular arrhythmogenesis even in this group, may be considered as new evidence that e-cigarette is not harmless as perceived by users. Widespread use of e-cigarette among youth, marketing them as harmless, extensive variance among brands in terms of e-liquid content, having limited knowledge about longterm e-cigarette use, production of e-liquids in uncontrolled locations, and causing increased heart rate and ventricular repolarization parameters in a similar way to conventional cigarettes is a crucial threat in terms of public health.

## Limitations

Our study has some limitations. The main limitation of our study was the relatively small number of cases and its single-center study design. Our study was cross-sectional, however, long-term prospective studies are needed to clearly demonstrate the relationship between Tp-e interval and Tp-e/QT ratio and ventricular arrhythmia in e-cigarette users. In addition, manual measurement may have rendered the ECG results of the QT and Tp-e intervals insufficient. In high-resolution digital systems, measurements could have reduced the margin of error even further. E-cigarette usage patterns were based on declarations of users. Therefore, conventional smokers or concomitant smokers may not be excluded. Another limitation of our study was the lack of blood nicotine level testing.

## Conclusion

In this cross-sectional study, important evidence demonstrating that e-cigarette use is not harmless in adults using e-cigarette is presented in comparison to healthy control participants who do not use e-cigarettes. It has been determined that e-cigarette use causes adverse alterations in ventricular repolarization, which may be potential indicators of ventricular arrhythmogenesis.
